# Information transfer through food from parents to offspring in wild Javan gibbons

**DOI:** 10.1038/s41598-019-57021-6

**Published:** 2020-01-20

**Authors:** Yoonjung Yi, Yena Kim, Agus Hikmat, Jae C. Choe

**Affiliations:** 1 Laboratory of Behavioral Ecology, Interdisciplinary Program of EcoCreative, Ewha Womans University, Seoul, 03760 Republic of Korea; 2 Cognitive Psychology Unit, Institute of Psychology, Leiden University, 2333 AK Leiden, the Netherlands; 3 Department of Forest Resources Conservation and Ecotourism, Faculty of Forestry, Bogor Agricultural University, Bogor, 16680 West Java Indonesia; 4 Laboratory of Behavioral Ecology, Department of Life Sciences and Division of EcoScience, Ewha Womans University, Seoul, 03760 Republic of Korea

**Keywords:** Behavioural ecology, Animal behaviour

## Abstract

The adaptive functions of food transfer from parents to their offspring have been explained mainly by two mutually non-exclusive hypotheses: the nutritional and informational hypotheses. In this study, we examined the functions of food transfer in wild Javan gibbons (*Hylobates moloch*) by testing these hypotheses from both infants’ and mothers’ perspectives. We observed 83 cases of food solicitations that resulted in 54 occasions of food transfers in three groups over a 19-month period in Gunung Halimun-Salak National Park, Indonesia. Infants initiated all solicitations directed at their mothers with one solicitation towards a father. Food solicitation rate decreased as infant age increased and ceased before weaning. As predicted by the informational hypothesis, infants solicited more food items difficult to obtain and preferred by their parents. On the contrary to the nutritional hypothesis, infants solicited low-quality items more often than high-quality items. Mothers did not change probability of food transfer according to the food characteristics or infant age. Hence, our results suggest that the primary function of food transfer from mother to infant Javan gibbons seems to be information transfer rather than nutritional aids, similarly to great apes.

## Introduction

Although food sharing among unrelated adults is somewhat rare, parent-offspring food transfer is reported in majority of primate species^1,2^. Food transfer from parents to offspring has been principally explained as a result of kin selection. Transferred food provides the offspring with a direct benefit, which in return provides parents with an indirect fitness benefit of increased offspring survival^3^. The adaptive functions of food transfer to offspring have been mainly explained by two mutually non-exclusive hypotheses: the nutritional and informational hypotheses^4–6^. The nutritional hypothesis states that food transfer enables offspring to grow and wean faster through the provision of extra nutrients^7^. Thus, the main predictions of the nutritional hypothesis are that food transfer will be most frequent during the weaning period, and that nutritionally rich food items will be transferred more than nutritionally poor items. The informational hypothesis claims that food transfer allows the immature to acquire food processing skills or information about food items which they cannot access themselves^7^. Therefore, following the informational hypothesis, as infants become older and more skilled, food solicitation rates are predicted to decrease^8^. Moreover, according to the informational hypothesis, novel or difficult-to-access/process food items are more likely to be transferred, regardless of their nutritional value.

Empirical findings in support of the nutritional hypothesis have been mostly found in cooperatively breeding primate species, such as the callitrichids, including tamarins and marmosets^9–11^. In these species, females typically produce 2–3 litters and become pregnant while lactating, which poses a greater energetic burden for the females^12–14^. Callitrichid parents actively participate in care-giving and food-provisioning to offspring to facilitate weaning process, and thereby shift their investment (*e*.*g*. energetic burden of lactation) from current to future offspring. In such a case, reproductive benefits to the parents are likely to exceed the cost of food provisioning to infants^15,16^. As predicted by the nutritional hypothesis, food transfer in callitrichids intensifies during the weaning period^17,18^. However, the nutritional hypothesis does not seem to be able to explain food transfer behaviour among great apes. In great apes, additional nutrients provided by parents might provide relatively lower support compared to callitrichids, due to (1) the slow development and long weaning period of great apes (*e*.*g*. ca. 7 years in orangutans^19^), (2) the absence of paternal care and (3) almost absence of twinning. Instead, support for the informational hypothesis has been found in great apes^9,20,21^; food transfer seems to directly affect knowledge acquisition among immatures in the foraging context^2,8,22^. For instance, food solicitation and transfer ceases prior to weaning in chimpanzees^23,24^ and immature orangutans mostly solicit items that are difficult to process by themselves^8^.

Food preference of group members can be an important source of knowledge, as it often carries information about nutritional value or palatability^25–27^. Multiple studies in mammals, including primates have demonstrated that food preference is socially transmitted to naïve individuals^25,26,28–32^. For instance, in an experimental setting, wild grivet infants (*Chlorocebus aethiops*) follow maternal food preferences^27^. Therefore, it is likely that food transfer also enables immatures to acquire information on food preference through parental food transfer, especially when they have not yet started independent foraging for solid foods. However, most studies on food transfer, especially in wild animals, did not consider the food preference as an important factor in terms of informational benefits. Food preference in observational studies often defines preferred food as food items that are eaten more frequently than their relative abundance^33^. Following this definition, we considered the parental food preference as another potentially important factor for the informational hypothesis and predicted that preferred foods would be solicited more to facilitate information transfer.

To fully understand the functions of food transfer, it is important to consider its cost (*e*.*g*. immediate loss of foods) from the food possessors’ perspective. When the costs of food transfer outweigh the benefits for the food possessor to transfer food, conflicts between possessor and receiver such as resistance or agonistic displays can occur. For example, food solicitation by infants may not always result in food transfer due to the partial intolerance of parents^7^. Mothers can selectively allow infants’ food solicitation, as the cost of food transfer depends on the length of the investment period (*i*.*e*. the age up until infants get provisioned), the amount (*i*.*e*. the frequency of food transfer) or the value of parental investment (*i.e*. transfer of rare or valuable food items)^7,34^. For instance, wild orangutan mothers transfer food less as their offspring become older and more competent in acquiring their own food^8^. Moreover, solicitation for novel food items is rejected more often than solicitation for familiar food items in captive lion tamarins (*Leontopithecus* spp.)^6^.

To date, most studies on the functions of food transfer in primates have been conducted on New World monkeys and great apes, but not yet on lesser apes, despite their position as a phylogenetic bridge between great apes and other primates. Only, a handful of studies have been conducted on food transfer in gibbons, with small datasets^35–37^ and focus on more proximate factors, such as the effects of hunger or individual rank on food transfer^38^. Gibbons have a long developmental period, with weaning occurring at *ca*. 22 months^39–41^ and sexual maturity at 6–8 years^42^. Twinning and paternal care, such as carrying, are extremely rare in gibbons with few exceptions^43,44^. Contrary to most great ape and some monkey species, gibbons do not use tools and do not rely on extractive foraging skills^45^. However, given their complex diet and high experienced temporal and spatial variation in food availability, learning is expected to play an important role in the development of foraging strategies in gibbon infants^46–48^.

In this study, we examined the functions of food transfer in wild Javan gibbons (*Hylobates moloch*) by testing the nutritional and informational hypotheses. Given the long developmental period of Javan gibbons, we predicted that the informational hypothesis, but not the nutritional hypothesis would be supported. Specifically, we predicted that 1) food solicitation rates would decrease as infants get older and 2) infants would solicit food items that are difficult to access/process and that are preferred by parents more than other items. Since costs of maternal investment (*i*.*e*. food transfer) to their infants would increase as the infants get close to wean, we predicted that 3) mothers would become less tolerant toward their infants as infants get older. In the same manner, costs associated with food transfer would be higher for the high quality, difficult to access/process, and preferred food items. Therefore, we predicted that 4) mothers would less frequently allow their infants to acquire those items compared to low quality, easy to access/process, and non-preferred food items. Understanding the functions and mechanisms of food transfer in both infants’ and mothers’ perspective may shed light on the development of foraging skills and social learning in gibbons.

## Results

Gibbon mothers fed on 106 different food items (group A: *n* = 78, group B: *n* = 70, group S: *n* = 66) from 73 plant species, as well as invertebrates and water. Infants solicited only 20 food items (group A: *n* = 14, group B: *n* = 11, group S: *n* = 7). However, among those items solicited by infants, six food items (nine cases of infants’ food solicitation) were rarely eaten by mothers and thus not caught in the scan sampling data at 15-min intervals for mothers’ diet. This indicates that infants often solicited rare food items.

**Figure 1:**
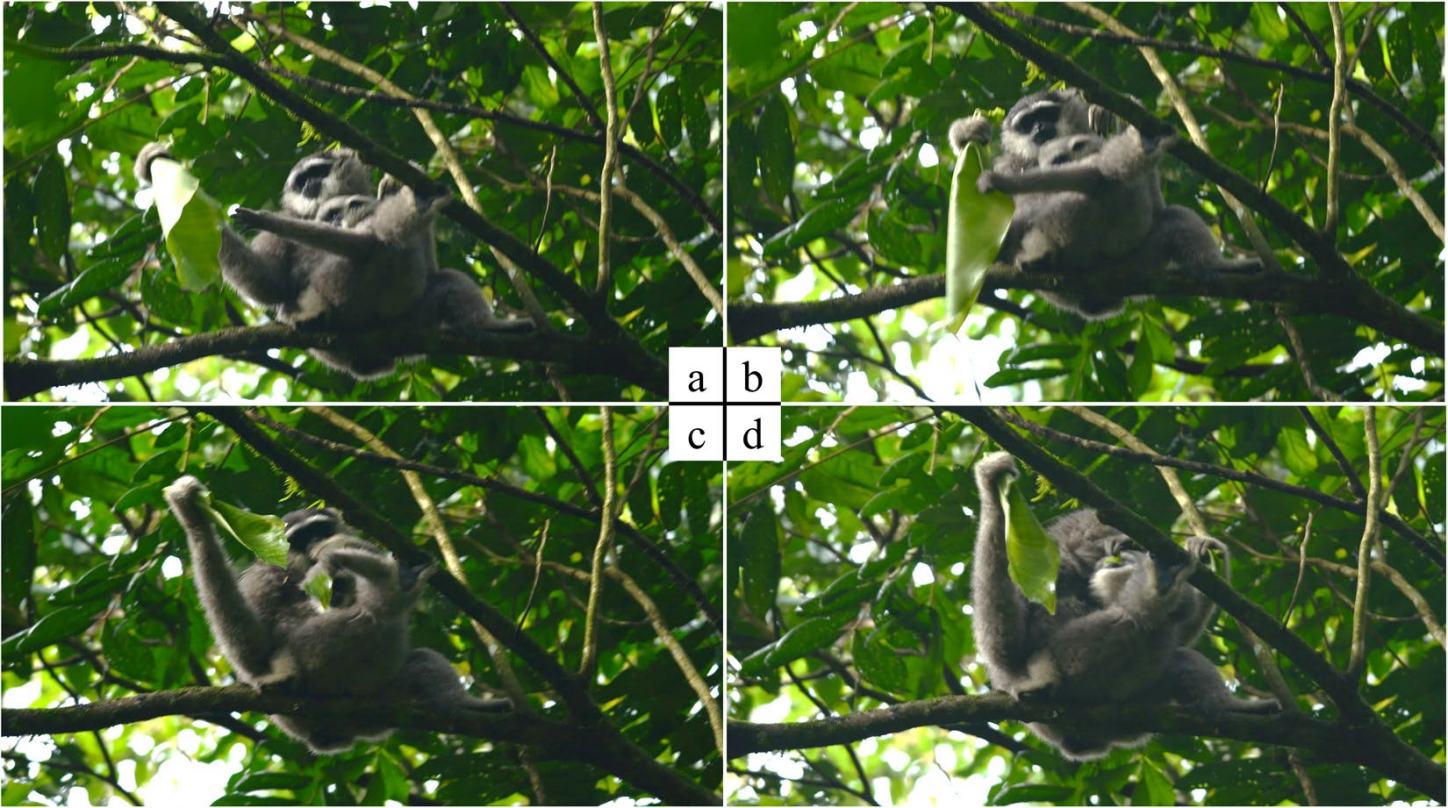
A case of food transfer from a mother to an infant Javan gibbon in Gunung Halimun-Salak National Park. (**a**) An infant stretched his arm toward a leaf that his mother was holding. (**b**) When the mother brought the leaf close to herself to consume it, the infant had the chance to grab the leaf. (**c**) The mother took the leaf away from the infant but the infant kept a part of it. (**d**) The infant also consumed the part of the leaf.

During the study period, we observed 54 cases of successful food transfer (group A: *n* = 21, group B: *n* = 19, group S: *n* = 14) from a total of 83 cases of food solicitation from the infants to their mothers (group A: *n* = 38, group B: *n* = 29, group S: *n* = 16; Fig. 1; Supplementary Table 1). The mean food solicitation frequency per hour was 0.034 (group A: 0.049, group B: 0.035, group S: 0.019). We also observed a single case of food solicitation towards a father in group A, which did not lead to a food transfer because of father’s rejection. Gibbon infants’ are known to start consuming solid foods at 4 months^49^. We have observed infants in our study site as well start consuming solid food at similar age, but nevertheless solicit food items that often can be obtained by themselves.

Among the 106 food items consumed by mothers, 11% (*n* = 12) were difficult food items, 68% (*n* = 73) were high-quality food items, and 15% (*n* = 16) were preferred food items. Among the 20 food items solicited by infants, 50% (*n* = 10) were difficult food items, 55% (*n* = 11) were high-quality food items, and 26% (*n* = 5) were preferred food items. Among 83 food solicitation events, infants solicited difficult food items in 75% (*n* = 62), high-quality food item in 55% (*n* = 46), and preferred food items in 35% (*n* = 27). Among 53 successful food solicitation events (*i*.*e*. food transfer), infants succeeded to acquire difficult food items in 79% (*n* = 43), high-quality food item in 53% (*n* = 29), and preferred food items in 33% (*n* = 16). For preference, we did not include solicitation of invertebrate (*n* = 5; all successfully transferred) since we do not have information on the mothers’ preference.

Additionally, we also observed mothers taking food from their infants six times. In two cases, mothers took the food back that had already been transferred to the infants within a minute. There was a single instance where a juvenile solicited food from its mother and it was rejected.

### Food solicitation: infants’ perspective

**Figure 2:**
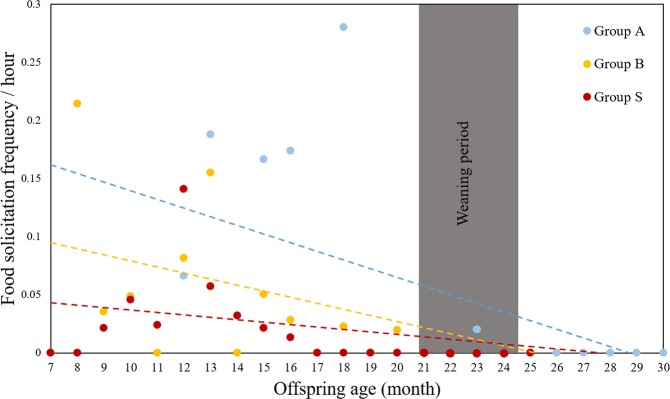
Food solicitation rate (solicitation frequency/observation hour) according to infant age (months) in Javan gibbons in Gunung Halimun-Salak National Park from November 2014 to July 2016. The dashed lines indicate the regression lines.

**Figure 3:**
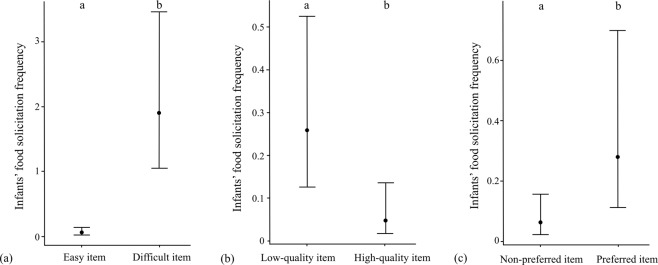
Infants’ food solicitation frequency for each food characteristic (**a**) Easy or difficult food item, (**b**) low-quality or high-quality food item, (**c**) non-preferred or preferred food item. Different letters on each boxplot indicate significant differences tested by the zero-inflated negative binomial model.

After infants presumably start consuming solid foods at around 4 months of their age, food solicitation rate peaked before weaning in group A and S, while the rate did not seem to have a clear peak in group B. The infants’ food solicitation ceased before weaning (at ca. 22 months), except for one solicitation event toward a father, which occurred at 23 months (Fig. 2). In general, infant age and food solicitation rates were negatively correlated (group A: *r* = −0.72, *n* = 19, *p* < 0.001; group B: *r* = −0.53, *n* = 19, *p* = 0.018; group S: *r* = −0.54, *n* = 19, *p* = 0.016). A zero-inflated negative binomial Generalized Linear Mixed Model (GLMM) with a log link function revealed that the difficulty, quality and preference of the food items significantly predicted the frequency of infants’ food solicitation (full-null model comparison: χ^2^ = 57.699, *df* = 3, *p* < 0.001). More specifically, difficult (*p* < 0.001), preferred (*p* = 0.014) and low-quality (*p* = 0.008) items were more likely to be solicited by infants (Table 1, Fig. 3). The proportion of mother’s foraging time on each food item did not affect the frequency of food solicitation by the infants (*p* = 0.363), suggesting that infants solicit food items selectively. However, the model was slightly overdispersed, with a dispersion parameter of 1.55 and therefore the results should be interpreted with a caution.

**Table 1:** Results of a zero-inflated negative binomial GLMM to test the effects of difficulty, quality, and preference of food items on the frequency of infants’ solicitation in three groups of Javan gibbons.

	Lower CI	Upper CI	Estimate	Std Error	z-value	*p*
Difficulty	2.722	4.262	3.492	0.393	8.892	<0.001***
Quality	−2.700	−0.401	−1.549	0.586	−2.645	0.008**
Preference	0.333	2.956	1.644	0.669	2.457	0.014*
Proportion of mothers’ foraging time^a^	−0.195	0.532	0.169	0.185	0.910	0.363

^*^<0.05; **<0.01; ***<0.001 – significance levels.

^a^z- transformed; mean ± SD of the original value: ^a^1.40 ± 2.19.

### Food transfer: mothers’ perspective

Infants failed to acquire food items in 29 of the total 83 food solicitation events, mostly due to the mothers’ active resistance by taking food items away from the infants’ reach. There was no proactive transfer from the parents, and all food transfers were initiated by the infants. Across the groups, on average, infants successfully acquired food in 69% of all solicitation events (group A = 52%, B = 66%, S = 88%). A binomial GLMM with a logit link function to determine the effects of infant age and food item characteristics (difficulty, quality, and preference) on the likelihood of the infants’ solicitation success was not significant (full-null model comparison: χ^2^ = 2.55, *df* = 4, *p* = 0.636). The results indicate that none of our variables could significantly explain the outcome of food solicitation.

## Discussion

The present study tested the functions of food transfer from Javan gibbon mothers to their offspring in the wild. Food transfer was relatively infrequent (mean food solicitation frequency/hour: 0.034), with only 83 cases of food solicitations resulting in 54 occasions of food transfers in three gibbon groups over a 19-month period. Infants’ food solicitation was always directed at their mothers, except one food solicitation event toward a father. Infants initiated all food solicitation and mothers have never voluntarily shared food with infants. We found no food solicitation or transfer between infants and older siblings. Moreover, we did not hear any specific vocalizations in food solicitation contexts either from mothers or infants, different from other species including primates^20,50–53^.

We investigated whether infants solicited food more as they grow older, and found that food solicitation rate ceased before weaning, following the informational hypothesis rather than the nutritional hypothesis (prediction 1). Although food solicitation rate increased presumably after the infants started to consume solid foods and peaked before weaning in two groups (group A and S), infant age and solicitation rate were in general negatively correlated. This suggests that the primary function of food transfer in Javan gibbons may not be nutritional aid which could facilitate growth or weaning. The result rather supports the informational hypothesis, which states that infants acquire necessary information about their diet through food transfer. Most mammals are required to reach sufficient levels of foraging competence at weaning to be able to independently forage and meet the nutritional requirement for body growth^54–58^, except few complicated skills such as tool-use or cooperative hunting^59^. Infant Javan gibbons start to consume solid foods at around 4 months of age^49^ and wean around 22 months^39–41^. No food solicitation by infants after weaning in Javan gibbons indicates that the infants have sufficient knowledge about their diet. We observed only a single case of a juvenile soliciting soil, a very rare food item from its mother during the whole study period. Javan gibbons at our study site consume ruddy soil which can only be found around the roots of an overturned tree (Yi, unpublished data). Since it requires them to climb down the tree and be on the ground, this behaviour seems particularly challenging for the immatures given their highly arboreal life style and the risk of exposure to ground predators. Thus, the ruddy soil could have been a novel item for the juvenile and by soliciting it may have gained informational benefits from the food transfer.

Infants mostly solicited food items that were difficult to obtain or that were preferred by their parents, following the prediction from the informational hypothesis (prediction 2). It is clear that the infants solicited difficult items a lot more than easy items, given the availability of the difficult items (12 out of 106 items) and the amount of solicited events directed to difficult items (62 out of 83 solicitations). Gibbons inhabit highly seasonal habitats with fluctuations in food availability, which forces them to develop foraging strategies to deal with its challenging environment^47,48^. However, it is also true that the diet of gibbons consists of relatively easy food items compared to that of other primate species that use extractive foraging skills, such as chimpanzees^60,61^, orangutans^62^, and capuchins^63^. Jaeggi and van Schaik^2^ argued that food sharing with offspring is significantly predicted by species’ extractive foraging skills for overall diet. However, immatures of the species which do not exhibit extensive extractive foraging can also have difficulty in accessing or processing some food items, because of their restricted locomotor ability and weaker grasping strength^49^. Also, their highly arboreal life sometimes requires gibbons to use only one hand to process the food, which poses the risk of falling when foraging at the end of the tree branches^64^. Therefore, it is highly likely that difficult food items still require the infants to learn specific knowledge and skills on food processing in Javan gibbons.

In this study, we considered parental food preference to also play an important role in infants’ social learning on diet, as it often carries information about the nutritional value and palatability^7,25–27^. Previous studies have shown that food solicitation by infants and transfer by adults increased for preferred food items in captive cotton-top and golden lion tamarins (*Saguinus oedipus*)^65^. Similarly, infants have been observed to solicit preferred food items more than other foods in captive gibbons^35,38^. Our results corroborate previous findings in captivity; parental food preference is a significant factor in predicting infants’ solicitation. Further studies should follow to better understand the role of food preference in terms of informational benefits and how food transfer can help infants to develop a food preference.

On the contrary, the predictions we tested did not seem to support the nutritional hypothesis in food transfer in Javan gibbons. Food solicitation generally decreased as the infants got older and ceased far before the weaning period where infants switch from milk to exclusively solid food and become independent from their mothers. Furthermore, high-quality food items were actually solicited less than low-quality items, even though the number of high quality items available was more than double the number of low quality food. Although it is likely that infant Javan gibbons also obtain nutritional benefits from the transferred food, the frequency and amount of the transferred food were only a fraction of the infants’ diets (54 instances of food transfer from all three gibbon groups over 19 months). The fact that food provisioned by family members can comprise up to 90% of infants’ diets in captive tamarins^17^ might indicate highly different functions of food transfer between callitrichids and apes.

Food transfer in Javan gibbons was relatively rare and always initiated by the infants, similar to the pattern found in the great apes (*Pan troglodytes*^24^, *Pongo pygmaeus*^8^). Oppositely, findings on callitrichids, parents proactively provision food to their infants and derive reproductive benefits from facilitated weaning^15,18^, which occurs relatively early (around 1–3 months)^10,15^. As Javan gibbons have relatively a long period of weaning and parental care is mostly restricted to mothers, it is unlikely that reproductive benefits of food transfer exceed their costs. To account for the mothers’ willingness to transfer food to their offspring in relation to the characteristics of food items and the infants’ age, we looked at the likelihood of successful food transfers on each solicitation event. We predicted increased conflicts over rare and valuable food items and increasing conflicts as offspring approach weaning age. However, opposite of what we predicted, Javan gibbon infant age did not explain the result of food solicitation (rejection of prediction 3), consistent with studies on great apes^23,24^. Also rejecting another prediction, tolerance of mothers (*i*.*e*. the result of food solicitation) did not differ according to the difficulty, quality, or preference of food items (rejection of prediction 4). A study in chimpanzees found similar patterns in which success rate did not differ across different foods^24^ (but see also^4,8^). The results can be related to parent-offspring conflict (POC)^34^ in food transfer context, indicating a low level of, or almost no POC in Javan gibbons. This might also be connected to the long developmental period in Javan gibbons, as a shorter developmental period often leads to the highly-concentrated POC^34^.

Moreover, we observed six cases of mothers taking food from their infants, but we did not observe any aggression in any of these cases. Nonetheless, during the study period we observed conflicts between parents and juveniles in a form of forced food taking by parents^66^. For example, mothers displaced their juvenile offspring from a big fig species, *Ficus punctata*, (difficult, high-quality, and preferred food) and consumed the left-over the juveniles were eating (Yi, unpublished data). This kind of aggression from mothers to offspring in the feeding context are common in primates^67^. The cases we report here can also be interpreted as juveniles turning into actual food competitors as they mature. However, it seems that the level of POC in Javan gibbons is generally low in the context of food transfer to infants.

Taken together, the results of our study suggest that the primary function of food transfer from parents to infants in wild Javan gibbons seems to support the information hypothesis similar to what has been found in great apes^5,21,24,68^. As suggested in other studies, food transfer can potentially facilitate the information transfer about food processing skills and knowledge on diets^6,69,70^. Our study has a limitation due to the small dataset to allow stable statistical analysis. However, food solicitation events were collected throughout the consecutive 19-month research period, using all-occurrence sampling. This implies that food transfer in Javan gibbons is not sufficient to serve for nutritional aids as the main function. In addition, significantly reduced food solicitation rates during juvenility might indicate increased knowledge and competence in acquiring and processing food for juveniles. Our study suggests that food transfer (aside from close-range watching of skilled individuals) is one of the most direct and critical ways through which immature primates learn the basics about their diets. Further studies on the direct link between immatures foraging behaviour and their past experiences in soliciting and receiving foods from their parents would help to uncover the role of food transfer in social learning.

## Methods

### Study species and site

This study was conducted in the Citalahab area (6.7412°S, 106.5309°E), a zone of primary forest in the Gunung Halimun-Salak National Park in Java, Indonesia. The study site is located 950–1,100 m above sea level, a relatively higher elevation than other habitats of Javan gibbons^71^. We studied three groups of Javan gibbons which were composed of an adult male-female pair with two to three offspring. We focused on the interactions between parents and the youngest offspring (infants) of the group, as food solicitation after infancy was very rare. We collected data from infant age of 7–25 month (group B and S) and 12–30 months (group A) covering most of infancy towards the weaning period at around 20–24 month of age. However, we also described rare cases of food solicitation or transfer between parents and older offspring (*i*.*e*. juveniles). We defined infants as 0–2 years, juveniles as 2–5 years, adolescents as 5–8 years, and sub-adults as +8 years, following the definition of age class in gibbons from Brockelman, *et al*.^41^.

### Data collection

We collected data from Dec. 2014 to Jun. 2016. We followed the gibbons all day from their sleeping tree to next sleeping tree. The total observation time was 2,209 h over 306 days (group A: 776.25 h over 105 days; group B: 720.5 h over 101 days; group S: 712.25 h over 100 days).

Food solicitation events between parents and offspring were collected using all occurrence sampling, while at least two observers always followed the focal individuals: adult females and infants for all social interactions^72^. For each food solicitation, we recorded the age of the infants (month), food items, and the result (success or failure). We encoded all food items into three binary categories following Silk (1978) and Nishida and Turner (1996): easy or difficult, high-quality or low-quality, and preferred or non-preferred. Thus, each food item was classified according to three characteristics (*e.g*. easy, low-quality, preferred). The following describes the definitions of the terms we employed:

#### Food item

Fruits, flowers, leaves, stems of a plant species, invertebrates, water and soil. We regarded each part to be different even for the same plant species since food items from one species can have different characteristics.

#### Food solicitation

Attempted acquisition of (or requesting for) food items by outstretching a hand towards a food item in the hand or mouth of a food possessor^24^. Outstretched hands towards the food possessors’ other body parts or hand/mouth without foods were not considered as food solicitation.

#### Food transfer

Any case where a part or a whole food item was transferred from one individual to another^73^. We used the term ‘food transfer’ instead of ‘food sharing’ throughout the study, to avoid implying any willingness to share by the initial food possessor^7^. Proactive food transfer refers to a food possessor actively initiate food transfer to another individual^74^.

#### Difficult and easy food

We followed Silk’s^4^ work and defined difficult food as food items that are difficult for infants to obtain independently. We considered a food item to be ‘difficult’ for Javan gibbon infants in our study site following five categories (Supplementary Table 2): (1) food items with thick shells (requiring extra manipulation), (2) food items growing on tree trunks (requiring the individual to be hanging on the branch with one arm while accessing the food items with the other arm; Supplementary Fig. 1), (3) food items on the ground (requiring the individual to climb down to the ground), (4) food items in tree holes (requiring the individual to put one arm deep inside to reach) and (5) invertebrates (requiring fine motor skill to catch moving prey). Hence, we categorized 6 fruits, 4 leaves, a stem, soil, water, and invertebrates as difficult food items, and the rest (*n* = 100) as easy food items.

#### High- and low-quality food

We followed definitions by Nishida and Turner^24^ and Jaeggi, *et al*.^8^, and regarded high-quality food as food items with high caloric value, such as fruits, flowers and invertebrates. Low-quality food items were leaves, stems, and soil (*cf*.^75^). Hence, we categorized 78 and 36 items as high and low quality food items, respectively.

#### Preferred food

Food items that were eaten more frequently than their relative availability^33^. We referred to the list of preferred food items from Kim, *et al*.^48^, a study conducted with the same study subjects in the same site. In contrast to other studies in gibbons, figs were not a fall-back, but were a preferred food of Javan gibbons in our study site^48^. We considered gibbons’ preference for invertebrates as unknown since we could not measure whether gibbons consumed invertebrates more than the relative amount available. Hence, we categorized 15 and 98 items as preferred and non-preferred food items, respectively.

We recorded all food species consumed by adult females using scan sampling at 15-minute intervals^72^ (N_foraging data point;_ group A = 803, group B = 661, group S = 675), which was collected over a consecutive 19-month period and therefore represents mothers’ diet from all year round. We used data from these scans to calculate the proportion of mother’s foraging time on each food item. We did not calculate the proportion of infant’s foraging time on each food item, since we did not collect scan sampling data at 15-min intervals from the infants.

### Data analysis

Statistical analyses are performed using the computing environment R^76^. To test whether solicitation rate decreases as the infants get older (prediction 1), we ran Spearman’s correlations between infant age (month) and monthly food solicitation rate for each infant. Monthly solicitation rate (*n* = 19) was obtained from the monthly food solicitation frequency divided by monthly observation time (hour).

Before fitting the models for the further analyses, we z-transformed numerical variables to facilitate model convergence. Random slopes were included to keep type I error rates at the nominal level of 5%^77^. We checked collinearity between factors using the R package ‘car’^78^ and the highest VIF value was 1.747. Furthermore we ran models with interactions first and then dropped the interactions when there was no significant effects. We did not find any significant effect of the interactions between factors, thus the interactions were dropped in all model. Full and null models were compared using a likelihood ratio test while null models only included control and random factors^79^. We also checked overdispersion for a zero-inflated negative binomial GLMM.

We performed a zero-inflated negative binomial GLMM using the package “glmmTMB”^80^ to test whether infants solicited specific food items (prediction 2). We used the frequency of infants’ solicitations by each food item for each group as a response variable. We excluded invertebrates from group A and S for the analysis, as we do not have information on gibbons’ preference on that food item, which resulted in the N_total_ = 212 (group A: *n* = 77, group B: *n* = 70, group S: *n* = 65). We included difficulty, quality and preference of food items as fixed factors to investigate the characteristics of food items solicited by infants. We also included the proportion of mothers’ foraging time on each food item as a fixed factor to control for the possible effect of infants soliciting food items that are more available than others, as a by-product of exposure time to the mother’s feeding. We included gibbon group ID as a random factor, and the proportion of mothers’ foraging time on each food item as a random slope within groups.

Finally, we performed a binomial GLMM to test whether the age of infants and food characteristics affected the result of infants’ food solicitation (i.e. whether mothers allowed them to take food items; prediction 3 and 4). We included the result of each food solicitation event (success vs. failure) as a responsible variable. We excluded infants’ solicitation on invertebrates (group A: *n *= 3, group B:* n* = 2) as we do not have information on gibbons’ preference on that food item, which resulted in N_total_ = 78 (group A: *n* = 35, group B: *n* = 27, group S: *n* = 16). We included the infant age (month), difficulty, quality, and preference of food items for each solicitation event as fixed factors. We included gibbon group ID as a random factor and the infant age as a random slope within group.

### Ethical note

We used non-invasive and behavioural observation data only. Our research protocol was approved by the Indonesian Ministry of Research and Technology (RISTEK), the Indonesian Ministry of Forestry’s Department for the Protection and Conservation of Nature (PHKA), and the Gunung Halimun-Salak National Park. Our research adhered to the American Society of Primatologist Principles of the Ethical Treatment of Non-Human Primates.

## Supplementary information

Supplementary Information.

## Data Availability

The datasets analyzed during the current study are available from the corresponding author on reasonable request.
