# Supplementary material for: Information transfer through food from parents to offspring in wild Javan gibbons
Source: Sci Rep. 2020 Jan 20;10:714. doi: 10.1038/s41598-019-57021-6 (PMC6971262; doi:10.1038/s41598-019-57021-6)

**Supplementary Information**

Information transfer through food from parents to offspring in wild Javan gibbons

Yoonjung Yi ^1^, Yena Kim ^2^, Agus Hikmat ^3^ and Jae C. Choe ^4^

^1^ Interdisciplinary Program of EcoCreative, Ewha Womans University, Seoul, 03760, Republic of Korea

^2^ Institute of Psychology, Cognitive Psychology Unit, Leiden University, 2333 AK Leiden, the Netherlands

^3^Department of Forest Resources Conservation and Ecotourism, Bogor Agricultural University, Bogor, 16680, West Java, Indonesia

^4^ Department of Life Sciences and Division of EcoScience, Ewha Womans University, Seoul, 03760, Republic of Korea

## Table S1. A list of food items solicited by infants of each gibbon group, and their solicitation frequency and characteristics.

## Table S2. A list of fourteen difficult food items defined in this study on Javan gibbons in Gunung Halimun-Salak National Park, Indonesia.

## Figure S1. A picture of *Ficus punctata*, the second most solicited food item by infant Javan gibbons in Gunung Halimun-Salak National Park (above). A gibbon has to be hanging to consume a fruit of *F. punctata* growing on tree trunk (below); therefore it has been categorized as a difficult food item.

Table S1.

| Gibbon group ID | Food item | Food part | Infant solicitation frequency | Difficulty | Quality | Preference |
| --- | --- | --- | --- | --- | --- | --- |
| A | Scindapsus marantaefolius | Leaf | 10 | Yes | No | No |
| A | Ficus punctata | Fruit | 8 | Yes | Yes | Yes |
| A | Sandorium Koetjapi | Fruit | 7 | Yes | Yes | Yes |
| A | Invertebrate | Live prey | 2 | Yes | Yes | Unknown |
| A | Water | Water | 2 | Yes | No | No |
| A | Artocarpus elasticus | Fruit | 1 | No | Yes | No |
| A | Bruinsmia styracoides | Fruit | 1 | Yes | Yes | No |
| A | Ficus padana | Fruit | 1 | No | Yes | No |
| A | Hoya macrophylla | Fruit | 1 | No | Yes | No |
| A | Knema cinerea | Fruit | 1 | No | Yes | Yes |
| A | Rhaphidophora pinnata | Leaf | 1 | Yes | No | No |
| A | Sandorium Koetjapi | Leaf | 1 | No | No | No |
| A | Scindapsus marantaefolius | Stem | 1 | Yes | No | No |
| A | Unknown | Stem | 1 | Unknown | No | Unknown |
| B | Scindapsus marantaefolius | Leaf | 9 | Yes | No | No |
| B | Hoya macrophylla | Fruit | 4 | No | Yes | No |
| B | Ficus punctata | Fruit | 3 | Yes | Yes | Yes |
| B | Invertebrate | Live prey | 3 | Yes | Yes | Unknown |
| B | Rhaphidophora pinnata | Leaf | 3 | Yes | No | No |
| B | Scindapsus marantaefolius | Stem | 2 | Yes | No | No |
| B | Ficus padana | Leaf | 1 | No | No | No |
| B | Garcinia dioica | Fruit | 1 | No | Yes | No |
| B | Melodinus orientalis | Fruit | 1 | Yes | Yes | No |
| B | Schefflera aromatica | Fruit | 1 | No | Yes | Yes |
| B | Soil | Soil | 1 | Yes | No | No |
| S | Scindapsus marantaefolius | Leaf | 6 | Yes | No | No |
| S | Ficus punctata | Fruit | 4 | Yes | Yes | Yes |
| S | Schefflera aromatica | Fruit | 2 | No | Yes | Yes |
| S | Artocarpus elasticus | Fruit | 1 | No | Yes | No |
| S | Ficus padana | Leaf | 1 | No | No | No |
| S | Nyssa Javanica | Fruit | 1 | No | Yes | Yes |
| S | Unknown | Fruit | 1 | Unknown | Yes | Unknown |

Table S2.

| No. | Scientific name | Local name | Food type | Categories of difficulty | Quality | Preference |
| --- | --- | --- | --- | --- | --- | --- |
| 1 | *Chrysophyllum roxburghii* | Culak ketan | fruit | thick shell | high | no |
| 2 | *Sandorium Koetjapi* | Kecapi | fruit | thick shell | high | yes |
| 3 | *Antidesma tetrandrum* | Kokosan monyet | fruit | thick shell | high | no |
| 4 | *Melodinus orientalis* | Liana enak | fruit | thick shell | high | no |
| 5 | *Ficus punctata* | Liana ficus besar | fruit | on tree trunk | high | yes |
| 6 | *Caryota mitis* | Sarai | fruit | on tree trunk | high | no |
| 7 | *Hoya macrophylla* | Liana daun tebal | leaf | on tree trunk | low | no |
| 8 | *Scindapsus marantaefolius* | Lolo | leaf | on tree trunk | low | no |
| 9 | *Oleandra pistillaris* | Pakis keras | leaf | on tree trunk | low | no |
| 10 | *Rhaphidophora pinnata* | Pakis liana | leaf | on tree trunk | low | no |
| 11 | *Scindapsus marantaefolius* | Lolo | stem | on tree trunk | low | no |
| 12 | *-* | Soil (red mud) | soil | on the ground | low | no |
| 13 | *-* | Water | water | in tree hole | low | no |
| 14 | *-* | Invertebrates | animal | alive | high | unknown |

Figure S1.


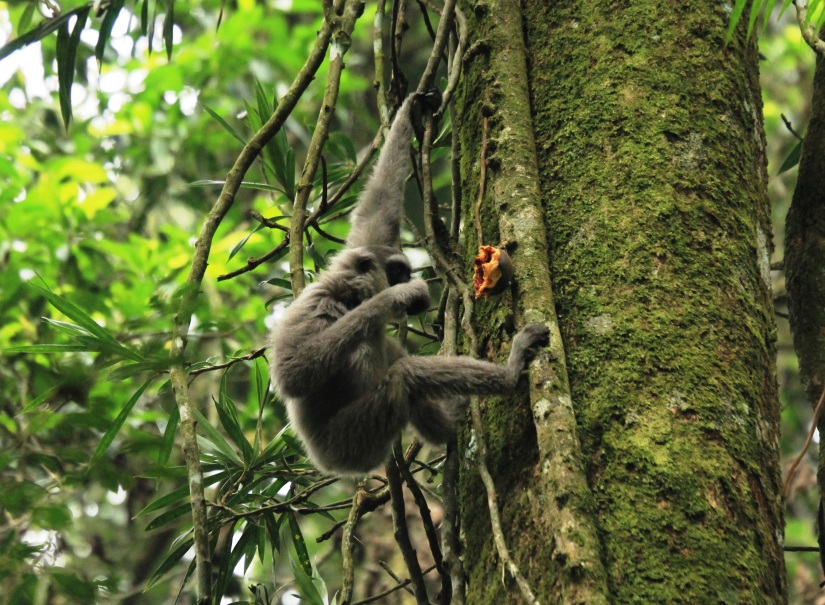

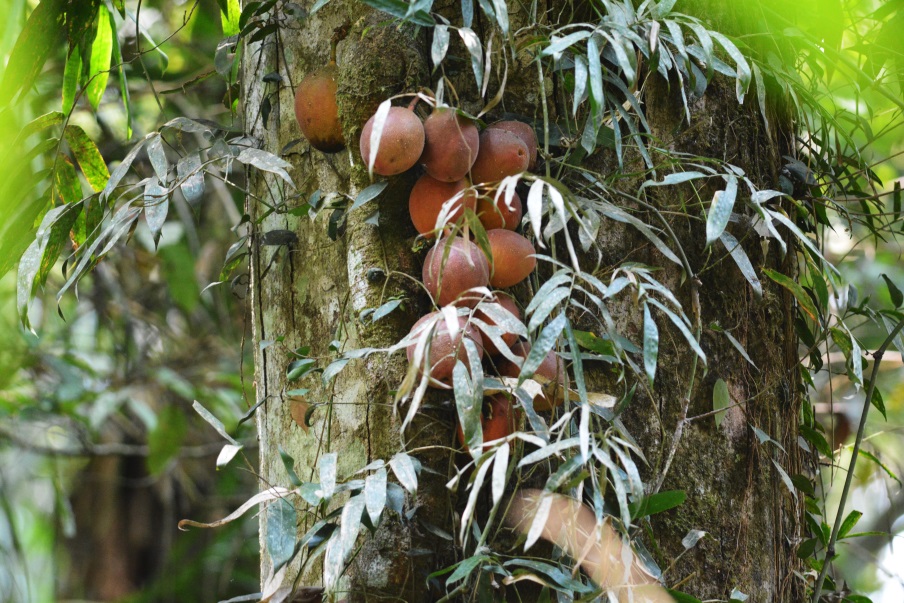

Supplement: Supplementary file 1 — Supplementary Information. [file 41598_2019_57021_MOESM1_ESM.docx]
